# Improved genome annotation through untargeted detection of pathway-specific metabolites

**DOI:** 10.1186/1471-2164-12-S1-S6

**Published:** 2011-06-15

**Authors:** Benjamin P Bowen, Curt R Fischer, Richard Baran, Jillian F Banfield, Trent Northen

**Affiliations:** 1 Department of GTL Bioenergy and Structural Biology, Life Sciences Division, Lawrence Berkeley National Laboratory, 1 Cyclotron Road, Berkeley, CA 94720, USA; 2 Department of Earth and Planetary Science, Policy, and Management, University of California at Berkeley, Berkeley CA 94720, USA; 3 Department of Environmental Science, Policy, and Management, University of California at Berkeley, Berkeley CA 94720, USA

## Abstract

**Background:**

Mass spectrometry-based metabolomics analyses have the potential to complement sequence-based methods of genome annotation, but only if raw mass spectral data can be linked to specific metabolic pathways. In untargeted metabolomics, the measured mass of a detected compound is used to define the location of the compound in chemical space, but uncertainties in mass measurements lead to "degeneracies" in chemical space since multiple chemical formulae correspond to the same measured mass. We compare two methods to eliminate these degeneracies. One method relies on natural isotopic abundances, and the other relies on the use of stable-isotope labeling (SIL) to directly determine C and N atom counts. Both depend on combinatorial explorations of the "chemical space" comprised of all possible chemical formulae comprised of biologically relevant chemical elements.

**Results:**

Of 1532 metabolic pathways curated in the MetaCyc database, 412 contain a metabolite having a chemical formula unique to that metabolic pathway. Thus, chemical formulae alone can suffice to infer the presence of some metabolic pathways. Of 248,928 unique chemical formulae selected from the PubChem database, more than 95% had at least one degeneracy on the basis of accurate mass information alone. Consideration of natural isotopic abundance reduced degeneracy to 64%, but mainly for formulae less than 500 Da in molecular weight, and only if the error in the relative isotopic peak intensity was less than 10%. Knowledge of exact C and N atom counts as determined by SIL enabled reduced degeneracy, allowing for determination of unique chemical formula for 55% of the PubChem formulae.

**Conclusions:**

To facilitate the assignment of chemical formulae to unknown mass-spectral features, profiling can be performed on cultures uniformly labeled with stable isotopes of nitrogen (^15^N) or carbon (^13^C). This makes it possible to accurately count the number of carbon and nitrogen atoms in each molecule, providing a robust means for reducing the degeneracy of chemical space and thus obtaining unique chemical formulae for features measured in untargeted metabolomics having a mass greater than 500 Da, with relative errors in measured isotopic peak intensity greater than 10%, and without the use of a chemical formula generator dependent on heuristic filtering. These chemical formulae can serve as indicators for the presence of particular metabolic pathways.

## Background

Untargeted profiling of small molecule metabolites using mass spectrometry has the potential to aid in the functional annotation of genomes. Comprehensive metabolite identification in untargeted metabolomics experiments would greatly improve downstream analyses, including metabolic network reconstruction [1,2] and metabolomics-aided genome annotation [3,4]. Specifically, detection of a compendium of metabolites in given organisms or communities can improve confidence in pathway-extension or hole-filing for sparsely annotated pathways [5-7]. In this manner, metabolomics provides an orthogonal resource that can complement sequence homology-based methods of genome annotation.

Identification of metabolites in untargeted mass spectrometry-based metabolomics using retention time, mass, and fragmentation pattern information remains a challenge [8], and validation of possible identifications by comparison to commercially available chemical standards is only possible for a subset of cases [9]. *De novo* identification of metabolites from spectral features or fragmentation (MS/MS) spectra is a tedious process and is currently not reliably scalable to large experiments [10]. However, the identification of a metabolite's chemical formula is a more tractable challenge, and formula assignment provides partial information about the identity of the observed metabolite. Typically, mass alone is not sufficient to specify the chemical formula [11,12].

The most common approach begins with combinatorial generation of possible chemical formulae that might correspond to a detected mass spectral feature. The astronomical number of possible formulae means that heuristic limitations are required to guide this combinatorial search. The most common restriction is to limit the elements that might comprise a detected ion to only those that are most biologically relevant: carbon, hydrogen, nitrogen, oxygen, sulfur, and phosphorus. Thus, formula generators must explore all possible formulae of the form C*_a_*H*_b_*N*_c_*O*_x_*S*_y_*P*_z_*, which spans a six dimensional space, where the dimensions are *a*, *b*, *c*, *x*, *y*, and *z*. For small molecule metabolites, maximal values for these dimensions might be close to 200 carbons and hydrogens, and lesser numbers of heteroatoms (see Materials and Methods), which still allows for a search space of 288,120,000 possible formulae.

Further heuristic restrictions, for example based valence requirements, have been used in some formula generating algorithms [11,12]. Relative isotope abundance patterns are reproducible and can be used to constrain likely chemical formula [13-15]. However, even when using restricted chemical formulae and isotopic data, the degeneracy around a mass value can still be high. A conceptual way to understand this point is to view mass as a single-dimensional projection of the six dimensional chemical space. Other information embedded in mass spectral data can serve as non-mass-based criteria to restrict the range of possible chemical formulae. The development of certain heuristics for prioritizing the likelihood of chemical formulae reduces the number of possible chemical formulae, but leaves some ambiguity that can be reduced through additional experimentation [11,12].

Modern mass spectrometers can constrain compound masses to within a few parts per million (ppm). Such accurate measurements assist in the task of determining chemical formulae (e.g., time-of-flight, ion-trap, and ion cyclotron resonance (ICR) mass spectrometers), especially when the mass of the target compound is large. Fourier transform ICR (FT-ICR) mass spectrometers have sufficient mass resolution and accuracy to enable use of isotopic fine structure for direct formula assignment. However, the majority of instruments used for untargeted metabolomics do not have such high resolution. In addition, to accurate mass measurements, accurate measurements of isotopic peak intensities are critical if natural isotopic abundance information is to be used. The importance of accurate intensity information increases as the mass of the target compound increases.

Notably, the use of stable isotope labeling has been shown to reduce the ambiguity of chemical formula assignment and has tremendous potential to aid in the comprehensive profiling of small molecules to better understand physiology [16-19]. Stable isotope labeling methods allow counting of C and N per formula unit and can lead to identification of the chemical formula without reliance on the natural isotopic abundance patterns and without using a restricted chemical formula generator.

In the current study, we compare chemical formula identification using natural isotopic abundance patterns to stable isotope labeling methods. We compare direct measurement of the counts of carbon and nitrogen atoms in an empirical formula to natural isotopic abundance information as a way to restrict chemical formula assignment. In addition, we show that simply identifying chemical formulae is sufficient to infer biological pathways. Thus untargeted metabolomics studies can inform genome annotation.

## Results and discussion

### Linking empirical formulas to metabolic pathways

**Figure 1:**
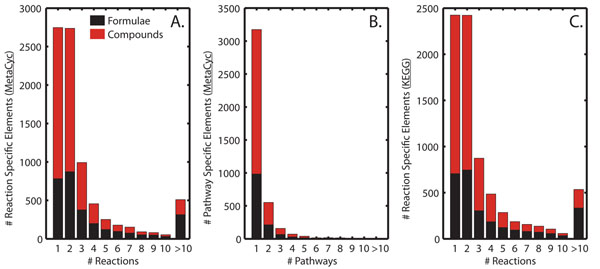
**Numbers of metabolites (red bars) and chemical formulae (black bars) in the MetaCyc database present only in a specific number of reactions (A) or pathways (B). Numbers of metabolites (red bars) and chemical formulae (black bars) in the KEGG database present only in a specific number of reactions (C). **Metabolites present in reactions not linked to any pathway in MetaCyc were not taken into account for panel B. A large number of metabolites and chemical formulae are unique, thus are associated with a single reaction or pathway.

To test the hypothesis that empirical formulae alone could pinpoint the presence of a particular metabolic pathway, we examined the MetaCyc collection of pathways and metabolites [20]. This analysis showed that many unique (not present in any other pathway or reaction not related to a pathway) metabolites and compounds with unique chemical formulae participate in a small number of reactions or pathways (Figure 1). Taking into account metabolites consisting of C, H, N, O, S, and P, there are 1532 pathways in MetaCyc that are not “Super-Pathways” (aggregates of multiple pathways). Of these pathways, 721 have at least one unique metabolite (Additional File 1). Additionally, 412 of these pathways have at least one metabolite with a unique chemical formula (Additional File 1). Identification of specific metabolites or merely the chemical formulae of detected metabolites may thus indicate the presence of specific reactions or pathways. This evidence cannot be considered conclusive, as specific metabolites can participate in reactions or pathways not covered by MetaCyc. Also, specific chemical formulae may correspond to metabolites not covered by MetaCyc or any known biological database. That said, mapping sets of experimentally identified chemical formulae on to genome-scale metabolic network reconstructions or databases of metabolism offers an attractive, first step approach for the evaluation of the quality of genome annotation. The approach can also highlight gaps in the annotation when unexpected metabolites are identified [16-19].

### Chemical degeneracy around a local mass-value

**Figure 2:**
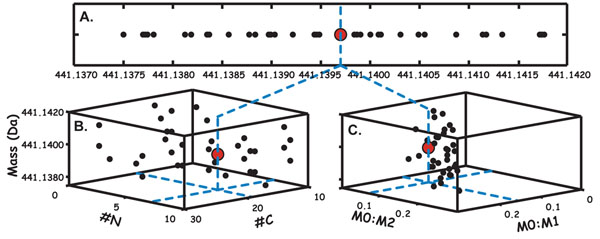
**Schematic illustrating conceptual and experimental approaches to representing and searching through chemical space. **As shown in (A), the monoisotopic mass of folate (M0) is projected into the range of possible chemical species in chemical space. Many distinct points in chemical space are nearly indistinguishable in this projection (“degeneracy”). In (B), the points are projected into 3 dimensional space where the number of nitrogen and carbon atoms in each chemical formula is known, and in (C), the isotopic intensities of M1 and M2 peaks relative to the M0 peak form other axes along which formulae can be projected.

Shown in Figure 2 are two approaches for reducing chemical degeneracy around an observed value in chemical space. High mass accuracy mass spectrometers achieve an uncertainty of approximately 5 ppm . Because of this uncertainty, there can often be a range of chemical formulae that could correspond to an "inexact mass". To demonstrate, the HR2 chemical formula generator was used to find points in chemical space that are within 5 ppm of folate (441.1397 Da) (Figure 2A) [12]. Due to the large number of possible chemical formulae associated with this mass value, biological inference is impractical. However, the ratio of the monoisotopic peak intensity (M0) to the peaks with 1 (M1) or 2 (M2) more neutrons can be used to prioritize the likelihood that points in chemical space correspond to the measured mass (Figure 2C). This widely used approach is described in the Seven Golden Rules. Alternatively, knowledge of the number of carbon atoms and nitrogen atoms can be used to reduce the number of chemical formulae in the search space (Figure 2B).

### Chemical formula generation as the first step in restricting chemical space

**Figure 3:**
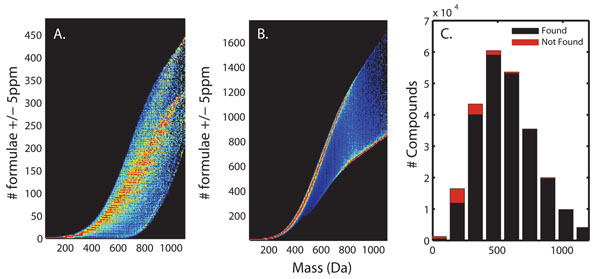
**Comparison of formula generation algorithms. **The HR2 algorithm (A) or the Brute Force algorithm (B) was used to estimate the mass degeneracy (to within 5 ppm mass accuracy) of representative points in chemical space, i.e., the number of unique chemical formulae within 5 ppm of a target mass. Brute force consistently found a higher mass degeneracy in chemical space (i.e., more possible formulae) than HR2. Additionally, for approximately 5% of the representative points chosen, HR2 (C) was unable to find any point in chemical space (i.e. recapitulate the formula corresponding exactly to the seed mass).

In Figure 3A, HR2 (with restrictions on allowable element ratios) was used to estimate the degeneracy in mass at 5 ppm for a library of unique mass values. In addition, Figure 3B shows the increase in degeneracy for an unfiltered (brute force) formula generator. The restrictions reduce the degeneracy, but some compounds are lost when formula generators are restricted by heuristics such as Lewis senior rules and ring/double bond equivalents, and allowable element ratios. In both cases, the degeneracy becomes unmanageable as the mass of a compound increases.

Accurate mass alone is insufficient to identify the chemical formula for high mass metabolites. The degree to which degeneracy increases with mass was evaluated for 248,928 compouds, each having a unique mass. These masses were selected from the PubChem database by including only chemical compounds comprised of less than 201, 201, 7, 21, 7, and 7 (respectively) atoms of the elements C, H, N, O, S, and P and having a mass of less than 1244 Da. The formulae of all these compounds could be generated by brute force. HR2, by design, uses heuristic filters to reduce the chemical formula search space; and therefore, it did not generate 11,380 of these formulae (Figure 3C). Most of these are unlikely to be biologically important (e.g. buckyballs: C_60_, tetrazete: N_4_). However, others, including ATP, taurine, and malate are of biological importance. These compounds are excluded by the compiled version of HR2 by restricting the oxygen to carbon ratio. This variable can be easily changed in the source code of HR2 to a more liberal value as described in the Seven Golden Rules [12]. We conclude that some metabolomics experiments can benefit from a less restricted formula generator, though use of an unrestricted chemical formula generator greatly increases the search space around a mass value. It is important to note that in cases where a compound has constitutional or stereoisomers and therefore lacks an unique chemical formula, the formula can provide valuable information to narrow the search, often to a given class of compounds (e.g. hexose), providing biological considerations.

### Defined C & N atom count for identification of a unique chemical formula

**Figure 4:**
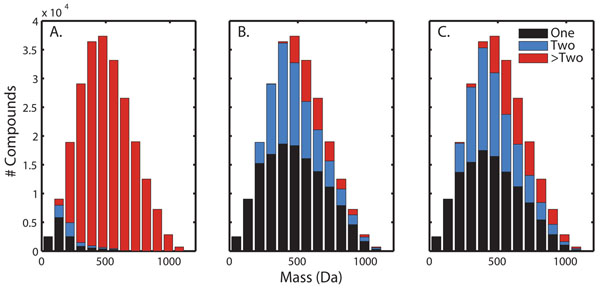
**Stable isotope labeling restricts the number of possible chemical formulae for measured mass values in metabolomics datasets. **All panels show a distribution of chemically representative unique masses in chemical space. In panel (A) HR2 was used to calculate the proportion of these unique masses having either no mass degeneracy (black) within (at 5 ppm), masses having a single mass degeneracy (blue), or two or more mass degeneracies (red). Panels (B) and (C) show how specifying points in chemical space by not only the exact mass, but also C atom counts and N atom counts as determined by stable isotopic labeling, affects the degeneracy. Panel (B) uses HR2 to estimate the degeneracy, while in (C) Brute Force is used.

By determining two dimensions in chemical space (the C and N count) the degeneracy of possible formulae is reduced. For the 248,928 chemically representative unique masses, we determined the degree to which specifying the C and N count determines a unique chemical formula (Figure 4). Using only knowledge from the HR2 chemical formula generator, unique mass defines chemical space location only for compounds less than approximately 500 Da (Fig 4A), and only for 5% of all compounds. When the unconstrained chemical formula generator (brute force) was applied to the same masses, very few could be localized to a specific coordinate in chemical space (0.5% of all compounds). However in Figure 4 B & C, determining only the number of carbon and nitrogen atoms for each mass reduces the degeneracy to a practical level (55% have a unique formula with HR2 and 50% with brute force).

### Comparison to relative isotopic peak intensities

**Figure 5:**
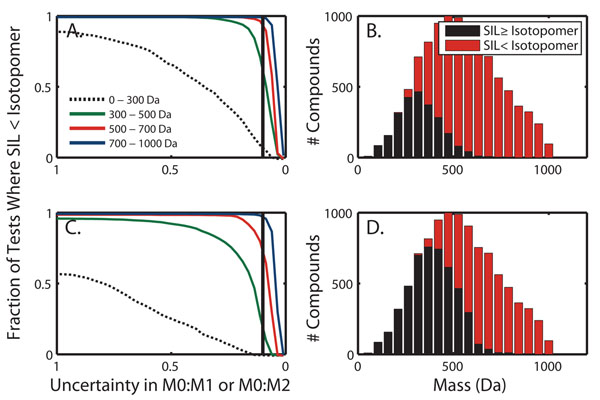
**Comparison of stable isotope labeling to relative isotopic peak intensity as a means of aiding unique formula determination. **Panels (A) & (C) show the fraction of tests where the specification of the chemical formulae using the C and N count was improved in comparison to using only relative isotopic peak intensity. Panels (B) & (D) show the mass distribution of tests where each method performed better or worse. Brute force was used in panels (A) and (B). HR2 was used in panels (C) and (D). These plots show that for larger compounds the C and N atom count provides information not obtainable from relative isotopic peak intensity.

The third of the Seven Golden Rules requires that the relative intensities of the peaks with one (M1) and two (M2) more neutrons compared to the monoisotopic (M0) peak are within a specified deviation compared to the pattern predicted for a chemical formula according to natural isotopic abundance. To determine the degree to which specifying the C and N count reduces degeneracy in comparison to this rule, 10,000 masses were selected at random from the library of 248,929 unique masses. For each selected mass value, formulae were generated within 5 ppm; and for each, the relative intensity of the M1 and M2 isotopic peaks as compared to the monoisotopic peak (M0) was calculated. This was done using both HR2 (Fig 5 C & D) and brute force (Fig 5 A & B). Furthermore, to test whether SIL knowledge was able to reduce the degeneracy better than the ratios of isotopic peak intensities, the number of possible chemical formulae remaining when the N and C count are known was also determined. This analysis was carried out as a function of the uncertainty in relative isotopic peak intensity. At zero-uncertainty, the ratio of the isotopic peaks uniquely defines a location in chemical space. However, realistically, there will be uncertainty associated with intensity [11,13]. At 10% uncertainty in the intensity ratios, the distribution of masses that were better localized by SIL or relative isotopic peak intensities is shown (Fig 5 B & D). Beyond approximately 500 Da, natural isotopic abundance information fails to achieve the improvement in chemical localization that SIL is capable of (typically a unique formula).

## Conclusions

While mass spectrometry alone often cannot determine which isomer of a metabolite is present, our analysis has shown that pathway-specific metabolites and metabolites with unique chemical formulae exist. Thus, if the entire spectrum of chemical formulae for an organism’s metabolites could be identified, clear designation of some metabolic pathways can be made.

To facilitate interpretation of metabolomics data, methods for identifying the chemical formula of detected features are greatly needed. A key deterrent to the identification of chemical formulae has historically stemmed from degeneracy, which increases with mass. We demonstrate here that the SIL method is better than existing methods at identification of chemical formulae for metabolites larger than 500 Da. This is achieved through determination of the C and N atom count. An additional advantage of the SIL method is that it functions well even when the relative error of the isotopic peak intensities is > 10%, however, this method has the disadvantage that it requires additional experimentation. We have shown that the use of heuristic filters in chemical formula generation, while effective at reducing degeneracy and do not require additional experiments, runs the risk of ignoring biologically relevant metabolites. This study demonstrates that the SIL method reduces degeneracy enough that unfiltered chemical formula generation is feasible.

## Methods

All figures and analyses were performed in Matlab 7.10.0 (R2010a) or Mathematica (v7.0.1).

### Identification of unique metabolites

MetaCyc version 14.1 was downloaded on 8/4/2010 [20]. The following files were used: compounds.dat, reactions.dat, and pathways.dat. From this, pathways which are not "Super-Pathways" were selected. All reactions and their corresponding metabolites containing only elements (C, H, N, O, S, and P) related to a pathway were identified. In total 8,741 metabolites were considered. When restricted by elements 7,782 remained. OF these, there were 4,178 unique formulae. Each metabolite in each pathway was examined to determine if the same metabolite or a complementary chemical formula was described in any other pathway or reaction not linked to a pathway.

### Generation of chemically representative unique masses

The PubChem database was downloaded on, October 6^th^, 2009[21]. Entries were imported with Mass ≥ 50 and ≤ 2000, not having non-natural isotopomers, and not having a charge explicitly stated in the molecular formula field (34,753,108 compounds). This list was then filtered to compounds that only have the following elements (C, H, N, O, S, and P), as these define the majority of biological metabolites (20,706,238 compounds). Further filtering to require (C, H, N, O, S, and P) to span the range of ([1:200], [1:200], [0:6], [0:20], [0:6], and [0:6]) respectively reduced the database size by 6.4%. Of the remaining 19,378,002 compounds, 248,928 have unique formulae. These chemically representative unique masses were used to perform the analysis presented here. Of the unique formulae in PubChem, 143,499 have a molecular weight greater than 500 Da. Although this ratio of heavy to light molecules is different than what would be found in MetaCyc (there are 1,833 out of 8,869 in MetaCyc that are between 500 and 2000 Da), the purpose in using PubChem is to attempt to explore a large chemical formula space.

### Comparison of formulae to those in the KEGG database

From ftp:kegg/compounds, a custom script was written to parse this file and return only those compounds that are not charged and have a defined chemical formula (not a polymer and not having a generic R-group) [22]. Out of 11,221 molecules, there are 6,181 unique chemical formulae. Of the unique chemical formulae, 5,042 are comprised of only CHNOPS and 5014 are within the 50 to 2000 Da mass range. There are 1,489 with a molecular weight greater than 500 Da.

### Determining the number of formulae within 5 ppm by HR2

The command line chemical formula generator was called for each of the unique masses described above[12]. The following string was issued to the program in order to constrain the possible formulae by the same constraints used for selecting the masses: "HR2-all-res.exe -C "test" -m MASS -t TOL -C 1-200 -H 1-200 -N 0-6 -O 0-20 -P 0-6 -S 0-6" where MASS is the neutral mass and TOL is the 5 ppm window size. The text output by HR2 was parsed using a custom script to return chemical formulae (additional file 2).

### Determining the number of formulae by brute force

A custom script was written in Matlab to generate all possible combinations over the range (C,H,N,O,S,P) of ([1:200], [1:200], [0:6], [0:20], [0:6], [0:6]) respectively. Formulae and corresponding masses within 5 ppm were returned.

### Isotopic pattern generator

A custom script was written in Matlab to generate relative isotopic peak intensities for a given chemical formula. The script uses multinomial probability distributions to calculate the exact abundance of the elemental isotopologues, one element at a time. The probabilities of a given isotomer for each element are binned on a user-defined mass-axis, and these vectors are then convolved to give the molecular isotopomer distribution patternthat includes all relevant elements.

## Competing interests

The authors declare that they have no competing interests.

## List of abbreviations used

SIL: Stable Isotope Labeling; PPM: parts per million; TOF: Time of Flight; HR2: A chemical formula generator constrained by heuristics [12].

## Author’s contributions

BB, RB, and CF developed the algorithms presented here. All authors contributed to experimental design and draft of the manuscript. All authors read and approved the final manuscript.

## Supplementary Material

Additional file 1 Table showing unique formulas and their pathways. Click here for file

Additional file 2 Table showing KEGG formulas not defined by HR2. Click here for file
